# Supplementary material for: Omnidirectional propulsion in a metachronal swimmer
Source: PLoS Comput Biol. 2023 Nov 17;19(11):e1010891. doi: 10.1371/journal.pcbi.1010891 (PMC10697607; doi:10.1371/journal.pcbi.1010891)
Supplement: S1 Text — The supporting text includes details for the solution procedure, the numerical implementation, the formulations for various coefficients, and the validation of the model against experimental data. (PDF) [file pcbi.1010891.s001.pdf]

Reduced-order swimming model

This supplementary material outlines the dynamics and solution procedure for the three-dimensional reduced-order ctenophore swimming model. Fig 1 shows the coordinate systems used to model the spheroidal body motion. We need two coordinate systems, an inertial system $\vec{x} = x_1\widehat{e_1} + x_2\widehat{e_2} + x_3\widehat{e_3}$, and a body-based coordinate system $\vec{x}' = x_1'\widehat{e_1'} + x_2'\widehat{e_2'} + x_3'\widehat{e_3'}$. As is typical in vehicle dynamics [1], we use the successive rotations (Z-Y-X or 3-2-1) yaw, pitch, and roll (ψ, θ, ϕ).

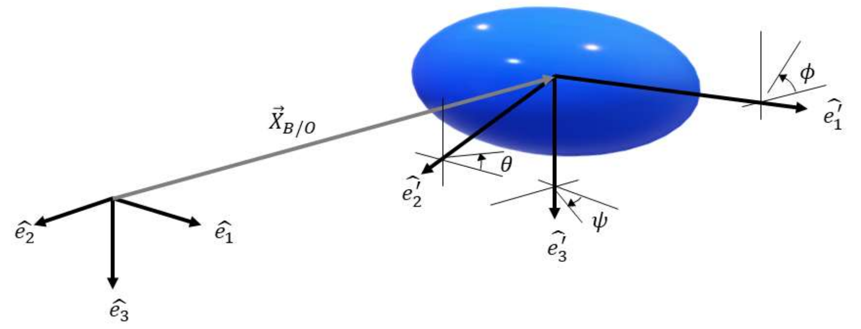

Fig S1. Schematic of a ctenophore's simplified geometry moving in a 3D space. The unit vectors $\widehat{e_1}$, $\widehat{e_2}$, and $\widehat{e_3}$ define the global (fixed) coordinate system while $\widehat{e_1'}$, $\widehat{e_2'}$, and $\widehat{e_3'}$correspond to the moving coordinate system attached to the spheroidal body.

The transformation between the inertial and body frames is given by $\vec{x}' = [C]\vec{x}$, where the transformation matrix is given by

$$[C] = \begin{bmatrix} \cos(\theta)\cos(\psi) & \cos(\theta)\sin(\psi) & -\sin(\theta) \\ \sin(\phi)\sin(\theta)\cos(\psi) - \cos(\phi)\sin(\psi) & \sin(\phi)\sin(\theta)\sin(\psi) + \cos(\phi)\cos(\psi) & \sin(\phi)\cos(\theta) \\ \cos(\phi)\sin(\theta)\cos(\psi) + \sin(\phi)\sin(\psi) & \cos(\phi)\sin(\theta)\sin(\psi) - \sin(\phi)\cos(\psi) & \cos(\phi)\cos(\theta) \end{bmatrix} \quad \text{(S.1)}$$

To avoid mathematical singularities when solving 3D motion using Euler angles (ψ, θ, ϕ), we performed all calculations using Euler parameters instead:

$$[C] = \begin{bmatrix} \beta_0^2 + \beta_1^2 - \beta_2^2 - \beta_3^2 & 2(\beta_1\beta_2 + \beta_0\beta_3) & 2(\beta_1\beta_3 - \beta_0\beta_2) \\ 2(\beta_1\beta_2 - \beta_0\beta_3) & \beta_0^2 - \beta_1^2 + \beta_2^2 - \beta_3^2 & 2(\beta_2\beta_3 + \beta_0\beta_1) \\ 2(\beta_1\beta_3 + \beta_0\beta_2) & 2(\beta_2\beta_3 - \beta_0\beta_1) & \beta_0^2 - \beta_1^2 - \beta_2^2 + \beta_3^2 \end{bmatrix} \quad \text{(S.2)}$$

The most general rigid body rotation has only three degrees of freedom; thus, the Euler parameters are subject to the constraint $\beta_0^2 + \beta_1^2 + \beta_2^2 + \beta_3^2 = 1$. To calculate the Euler parameters $(\vec{\beta})$, we use the Stanley method [1]. The last step for a formulation based on Euler parameters is to find the relationship between the time rates of change of $\vec{\beta}$ and the body angular velocities ($\vec{\omega}'$). This relationship is known as the Euler parameter kinematic differential equation:

$$\begin{bmatrix} \dot{\beta}_0 \\ \dot{\beta}_1 \\ \dot{\beta}_2 \\ \dot{\beta}_3 \end{bmatrix} = \begin{bmatrix} -\beta_1 & -\beta_2 & -\beta_3 \\ \beta_0 & -\beta_3 & \beta_2 \\ \beta_3 & \beta_0 & -\beta_1 \\ -\beta_2 & \beta_1 & \beta_0 \end{bmatrix} \begin{bmatrix} \omega_{x'} \\ \omega_{y'} \\ \omega_{z'} \end{bmatrix} \quad (S.3)$$

Hence, under an Euler parameter formulation, we have three (vector) governing equations: the first and second Euler's laws (equations (2) and (3) in the manuscript) and the Euler parameter kinematic differential equation (S.3).

Propulsion force $(\vec{F}_{net})$

As described in the main manuscript, each ctene is modeled as an oscillating flat plate, whose kinematics $(x_A(t), y_A(t))$ depend on the beating parameters and placement on the animal body (Fig 9 in the manuscript). Here we will expand on the formulations presented in the manuscript. Starting with the oscillating flat plate kinematics, by geometry, we can define the elliptical trajectory as a function of l, Φ, and Sa. Recall that $Sa = \frac{A_e}{A_o}$, where A_o is the area of the largest ellipse inscribed in the reachable space of the ctene (half circle with area $\pi l^2/2$, Fig 2A), and A_e the enclosed tip area, which for an ellipse equals πab, where a/b are the semimajor/minor axes.

From geometry, $A_o = 0.77\frac{\pi l^2}{2}$; from that, we calculate the semiminor axis as $b = 0.385\frac{l^2}{a}Sa$. As evident from Fig 2B, the semimajor axis is $a = l\ sin(\Phi/2)$. The center of the ellipse is $x_c = 0$ and $y_c = l\ cos(\Phi/2)$.

Now that we can describe the plate tip trajectory as a function of beating parameters, the kinematics are given by the parametric equations of the ellipse: $x_A = x_c + \mathrm{a}\cos(\theta)$, $y_A = y_c + \mathrm{b}\sin(\theta)$, $\dot{x}_A = -a\dot{\theta}\sin(\theta)$, and $\dot{y}_A = b\dot{\theta}\ cos(\theta)$. Where θ is the angle swept by the plate tip as it moves around the ellipse, which itself is a function of f and Ta. In this model, we assume constant angular velocities for the power and recovery strokes such that $\dot{\theta}_p = \frac{\pi}{t_p}$ and $\dot{\theta}_r = \frac{\pi}{t_r}$. The power and recovery stroke times can be calculated from the temporal asymmetry and the cycle period ($T = 1/f$) as $t_r = T(Ta + 1)/2$ and $t_p = T - t_r$. Finally, we continuously evaluate the swept angle $\theta(t)$ with the following piecewise-defined function:

$$\theta(t) = \begin{cases} \dot{\theta}_p[t - (mt_r)] + m\pi & for \quad mT \leq t \leq mT + t_p \\ (m-1)\pi + \dot{\theta}_r\left[t - (m+1)t_p\right] & for \quad mT + t_p < t \leq (m+1)T \end{cases} \qquad (S.4)$$

where m is the cycle number ($m = 0,1,2,3,\ldots$). These equations model the oscillating flat plate kinematics with respect to its central position O_A. The next step is to "place" them on the body.

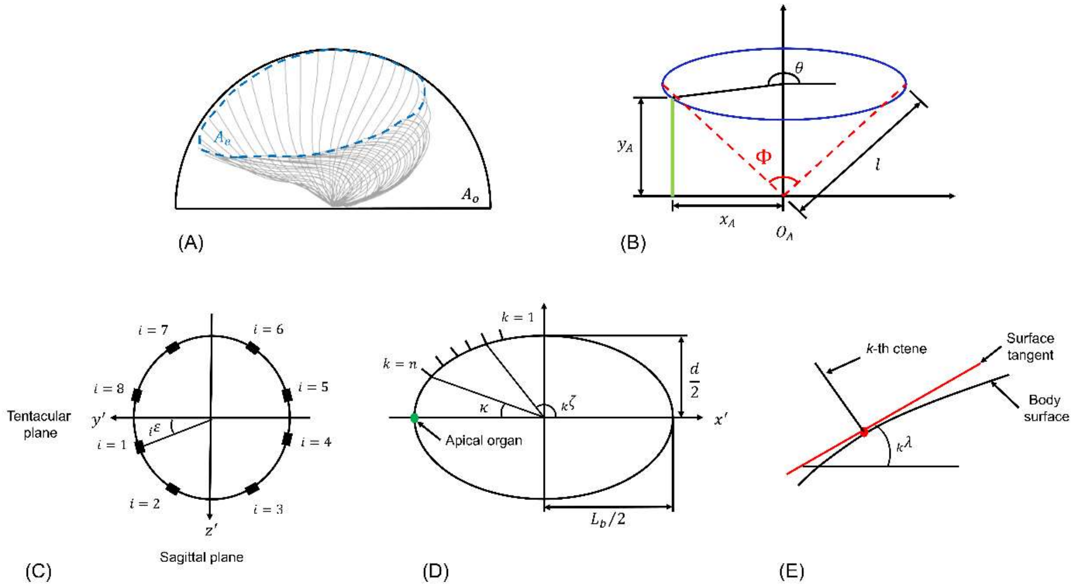

Fig S2. (A) Graphical description of the spatial asymmetry overlaid on the ctene lateral profile time series. (B) Simplified elliptical trajectory (blue line, (x_A, y_A)) and the oscillating flat plate (green line). Dotted red lines denote stroke amplitude (Φ), and l is the ctene length. (C) Top view of a modeled ctenophore, showing the tentacular and sagittal planes. Black boxes indicate the ith ctene row and $_i\varepsilon$ the corresponding ctene row position angle. (D) Side view showing a plane bisecting a ctene row. Black lines protruding from the body represent the kth ctene on the row. Ctene rows start at a fixed angle κ with respect to the apical organ, and $_k\zeta$ is the ctene positioning angle. (E) Close up of a ctene position (red dot), showing the tangential angle to the body surface $_k\lambda$.

To "place" each oscillating flat plate, we need three position angles: the ctene row angle $_i\varepsilon$, the ctene position angle on the ctene row $_k\zeta$, and the angle of the first ctene on the row κ (Fig 2C and D). These angles are based on measurements from experimentally observed animals: $_i\varepsilon$ and κ are measured directly, while $_k\zeta$ is determined based on the average spacing ratio $s = l/\delta$, where δ is the arc length of the body surface between ctenes (the perimeter of the modeled body is given by $a\int_0^{\zeta}\sqrt{1 - m\,sin^2(\zeta)d\zeta}$, where $m = 1 - (b/a)^2$). Finally, the plates oscillate tangentially to the

body surface (Fig 2E); the tangential angle is calculated from the parametric equations of the ellipse that defines the spheroidal body as ${}_{k}\lambda = tan^{-1}\left[\frac{-\mathrm{a\,c}\quad\left({}_{k}\zeta\right)}{b\sin\left({}_{k}\zeta\right)}\right]$.

Force and Torque coefficients

Drag coefficient for an oscillating flat plate

To model the drag of the oscillating plate while considering the correct Reynolds number range for ctene beating $(1 < Re < 200)$, we use the empirical expression obtained by [2], appropriate for Reynolds numbers between 1 and 1057.

$$C_A = 15Pe^{-0.5}\exp\left(\frac{1.88}{Re^{0.547}}\right) \qquad (S.5)$$

where Pe is the period parameter and is defined as $Pe = \left(\left|{}^{max}_{ik}\vec{u} - \dot{\vec{X}}_{B/0}\right| T\right)/w$, and the Reynolds number is defined as $Re = \left(\left|{}^{max}_{ik}\vec{u} - \dot{\vec{X}}_{B/0}\right| w\right)/\upsilon$.

Drag coefficient for a prolate spheroid

From [3], we obtain an expression for the drag coefficient of a prolate spheroid:

$$C_B = C_{shape}\left[\frac{24}{{Re_B}^*}\left(1 + 0.15{Re_B}^{*0.687}\right) + \frac{0.42}{1 + \frac{42{,}500}{{Re_B}^{*1.16}}}\right] \qquad (S.6)$$

$${Re_B}^* = \frac{C_{shape}}{f_{shape}}\left(\frac{\left|\dot{\vec{X}}_B\right| d_e}{\nu}\right)$$

$$C_{shape} = 1 + 0.7\sqrt{\left({A_{surf}}^* - 1\right)} + 2.4\left({A_{surf}}^* - 1\right)$$

$$A_{surf}^{\ *} = \frac{1}{2\alpha_s^{\frac{2}{3}}}\left[1 + \frac{\alpha_s}{\sqrt{1-\alpha_s^{-2}}} sin^{-1}\left(\sqrt{1-\alpha_s^{-2}}\right)\right]$$

where d_e is the spherical equivalent diameter and f_{shape} depends on flow direction

$$f_{sha}^{\parallel} \quad = \frac{(4/3)\alpha_s^{-\frac{1}{3}}(1-\alpha_s^{2})}{\alpha_s - \frac{(2\alpha_s^{2}-1)\ln\left(\alpha_s + \sqrt{\alpha_s^{2}-1}\right)}{\sqrt{\alpha_s^{2}-1}}}$$

$$f_{shape}^{\perp} = \frac{(8/3)\alpha_s^{-\frac{1}{3}}(\alpha_s^{2}-1)}{\alpha_s + \frac{(2\alpha_s^{2}-3)\ln\left(\alpha_s + \sqrt{\alpha_s^{2}-1}\right)}{\sqrt{\alpha_s^{2}-1}}}$$

where α_s is the aspect ratio of the body, $\alpha_s \equiv L_b/d$.

Added mass coefficients

From [4], we obtain the added mass coefficients for a spheroidal body for the axial and lateral movements (k_1, k_2):

$$k_1 = \frac{1-e^2}{e^3}\left[\ln\left(\frac{1+e}{1-e}\right) - 2e\right] \qquad (S.7a)$$

$$k_2 = \frac{1-e^2}{e^3}\left[\frac{e}{1-e^2} - \frac{1}{2}\ln\left(\frac{1+e}{1-e}\right)\right] \qquad (S.7b)$$

where e is the eccentricity $\left(e = \sqrt{b^2 - a^2/b^2}\right)$. This approach is fully valid only for linearly superposable flows (i.e. potential flow (high Re) or Stokes flow (low Re)) but is a good engineering approximation for intermediate Reynolds numbers [5].

Torque coefficients

To model the opposing torques, we used the numerical expressions obtained by [6], which is appropriate for rotating Reynolds numbers $\left(Re_R = \frac{d_e^2|\vec{\omega}'|}{\nu}\right)$, between $10^{-1} - 10^{3}$.

$$C_R = r_1(Re_R)^{r_2} + \frac{r_3}{(Re_R)^{r_4}} \qquad (S.8)$$

where the coefficients r_i depend on the rotating direction (rolling or pitch/yaw axes); see Table 1.

Table S1. Values for the torque coefficient expression along the roll and pitch/yaw directions

Turning axis	r_1	r_2	r_3	r_4
roll	0.573	−0.154	116.61	1
pitch/yaw	1.244	0.239	378.12	0.789

Solution procedure

We solved the reduced-order model using a fourth-order Runge-Kutta scheme for implicit equations, using the MATLAB function ode15i. The solution algorithm consists of the following steps:

1. Input the initial particle position, orientation, and speeds (translational and angular).
2. Calculate the initial transformation matrix using equation (S.1).
3. Calculate the corresponding initial values of the Euler parameters using the Stanley method and evaluate the transformation matrix in its parametrized form using equation (S.2).
4. Evaluate the propulsion and opposing forces and torques (equations (6), (7), (8), (9), and (10), in the manuscript) at the current time instant for all the (predetermined) morphometric and metachronal parameters.
5. Solve the equations of motion as given by equations (2), (3), and (S.3).

6. Return to step 4 and continue until the halting condition is met. Validations and Motor Volume calculations are halted after a certain solution time; while the MAP results are halted when a steady state radius of curvature is achieved

The numerical integration of the equations of motion iterated until it reached tolerances of 10^{-5}.

Swimming model verification

To confirm that our reduced-order model can estimate the forces and torques present in ctenophore swimming, we will compare the model predictions to freely swimming ctenophores. We considered two experimentally observed turning sequences: 1) mode 1, with only two active ctene rows, and 2) mode 3, with 6 rows beating at a higher frequency. According to our definition of beat frequency ($f = 1/T$, where T is the time between two power strokes), the animals can change the beat frequency for each beat cycle; however, the reduced-order model requires a frequency input at a higher time resolution. Fig 3 shows the observed beat frequencies for the two active ctene rows for Sequence 1, measured by counting the beat period of ctenes on the three camera views (blue dots). We artificially increased the time resolution by fitting a spline to the measured beat frequencies (black line). This approach results in a continuously varying paddle speed instead of the discrete beat frequency of the animals; however, it also smooths out the rapid oscillations that appear to be present (see Fig. 3) in the recorded sequence. We note that these oscillations are largely an artefact of the way we have defined frequency, which dictate that it can only change once per beat cycle). This ensures that overall swimming trajectories remain smooth, but may miss any physical consequences of these apparent rapid changes in frequency.

The beat sequence for mode 1 has $\overline{R/L} = 0.13$ and $\bar{V} = 0.4\ BL/s$. We run our reduced-order swimming model based on these observations and the morphometrics reported in Table 2. Fig 4A

compares the experimental (red line) and predicted (blue line) swimming trajectory. As explained in the manuscript, available camera resolution precludes simultaneous measurement of the ctene-level spatiotemporal asymmetries together with trajectory tracking; thus, the shaded areas in Fig 4A show the entire solution space ($0.1 < Sa < 0.6$ and $0.1 < Ta < 0.6$), and the blue solid line is the best prediction for this case (taking $Sa = 0.2$ and $Ta = 0.6$, both reasonable values). Fig 4B shows the experimental tracked triangle (red, formed by the tentacular bulbs and the apical organ; see Fig 1 of the manuscript) *vs.* and the predicted triangle (blue) for different timepoints ($t = 0, 1.5, and\ 2.5\ s$). The agreement between the experimental and predicted triangles shows the model capacity to predict the orientation of the animal.

For the second case (mode 3), Fig 5 shows the beat frequencies for each of the eight ctene rows. Here turning is powered mainly by ctene rows 7 and 8 (Fig 3H and 3I), which beat with a lower frequency for the first second. This results in $\overline{R/L} = 0.08$ and $\bar{V} = 0.24\ BL/s$. Fig 6A compares the experimental (red line) and predicted (blue line) swimming trajectory, and the shaded areas in Fig 6A show the entire solution space ($0.1 < Sa < 0.6$ and $0.1 < Ta < 0.6$). For this case, the best prediction (blue line) has asymmetry values of $Sa = 0.18$ and $Ta = 0.18$. Fig 6B shows the experimental tracked triangle (red), and the predicted triangle (blue) for different timepoints ($t = 0, 1.5, and\ 3\ s$).

Both comparisons show that our highly simplified mathematical model can predict propulsion and opposing forces/torques similar to those experienced by a swimming ctenophore. Therefore, we are justified in using this model for our parametric exploration of the maneuverability and agility of the ctenophore body plan and locomotion strategy.

Table S2. Morphometric measurements of observed animals

Maneuver	$L_B(mm)$	$d_B(mm)$	$l(mm)$	s	n_S	n_T
Mode 1	7.42	7.68	0.43	0.6	10	9
Mode 2	8	5.07	0.47	1.2	9	7

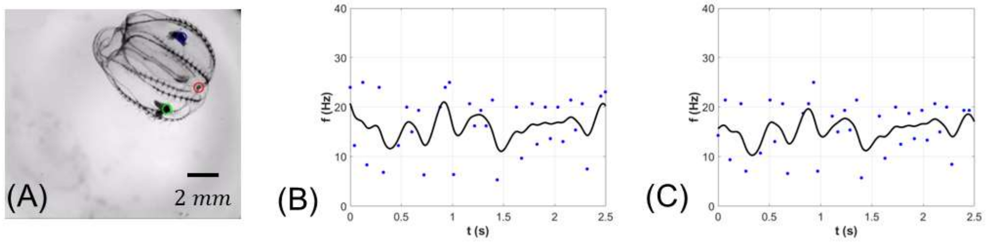

Fig S3. Beat frequency measurements for the mode 1 turning trajectory. (A) Snapshot of freely swimming ctenophore and the tracked points: apical organ (red) and tentacular bulbs (blue and green). (B) and (C) show the direct frequency measurements for ctene rows 4 and 5, respectively (bottom ctene rows). Dots represent measurements, and the fitted black line is used as an input to calculate the kinematics of the oscillating plates in the mathematical model.

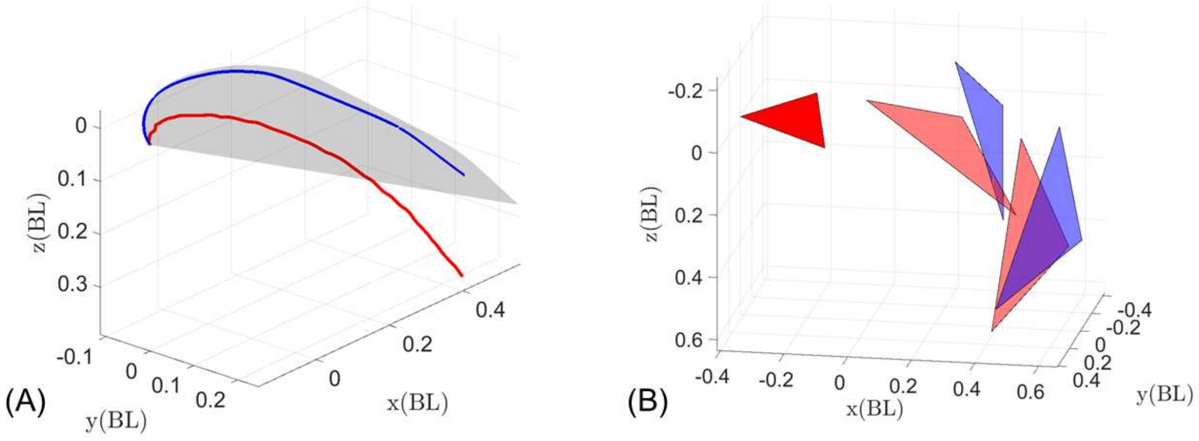

Fig S4. Comparison between experimental measurements (red) and mathematical predictions (blue) for the mode 1 turning trajectory. (A) shows experimental vs predicted swimming trajectories. The shaded area shows the entire spatiotemporal solution space ($Sa - Ta$), while the blue line is the best model prediction ($Sa = 0.2$ and $Ta = 0.6$). (B) shows experimental vs predicted swimming orientation. The red triangles show the experimental positions for the tentacular bulbs and the apical organ for different timepoints t = 0, 1.5, and 2.5s. The blue triangles are the best fit ($Sa = 0.2$ and $Ta = 0.6$) predicted positions for the same time instants.

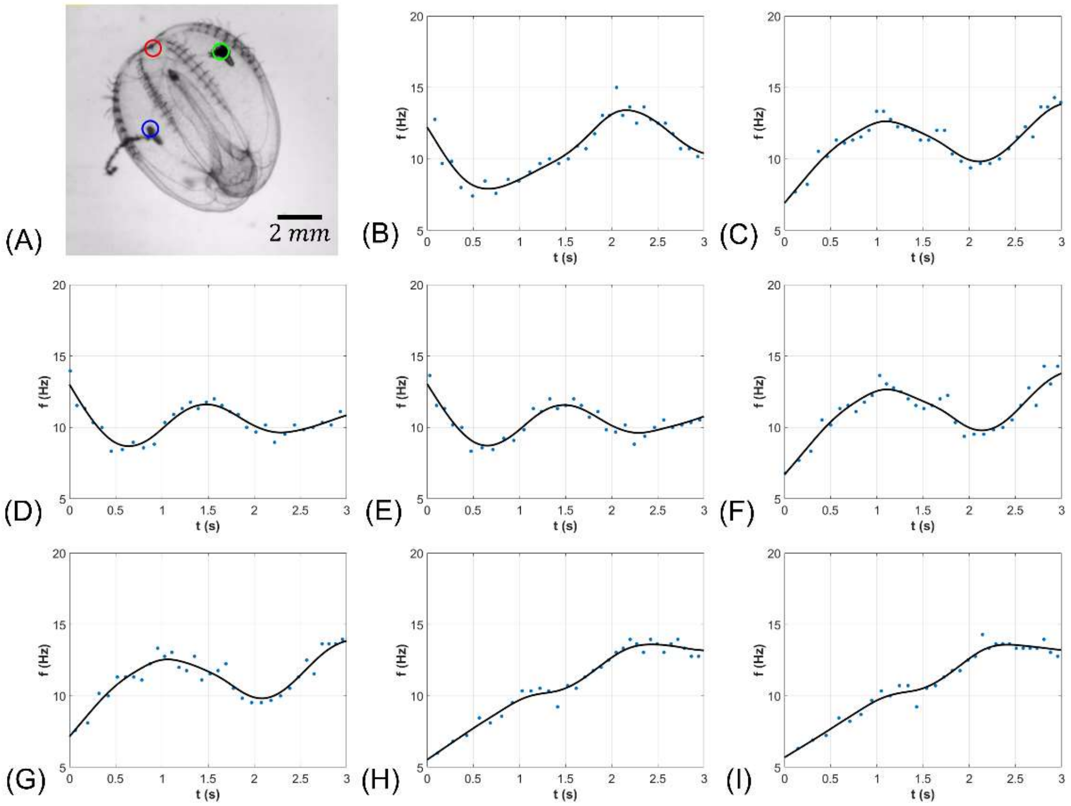

Fig S5. Beat frequency measurements for the mode 3 turning trajectory. (A) Snapshot of freely swimming ctenophore and the tracked points: apical organ (red) and tentacular bulbs (blue and green). (B) to (I) show the direct frequency measurements for ctene rows 1 to 8. Dots represent measurements, and the fitted black line is used as an input to calculate the kinematics of the oscillating plates in the mathematical model.

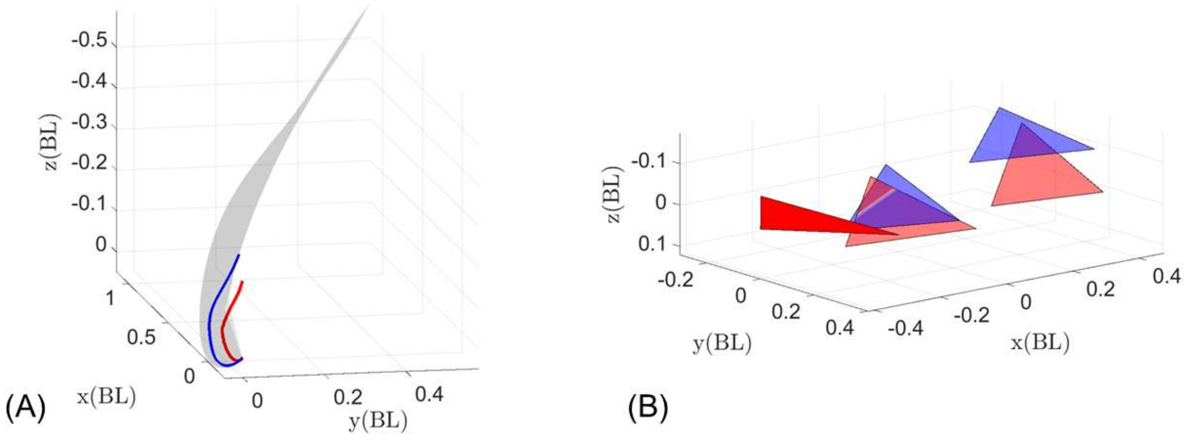

Fig S6. Comparison between experimental measurements (red) and mathematical predictions (blue) for the mode 3 turning trajectory. (A) shows experimental vs predicted swimming trajectories. The shaded area shows the entire spatiotemporal solution space ($Sa - Ta$), while the blue line is the best model prediction ($Sa = 0.18$ and $Ta = 0.18$). (B) shows experimental vs predicted swimming orientation. The red triangles show the experimental positions for the tentacular bulbs and the apical organ for different timepointst = 0, 1.5, and 3s. The blue triangles are the best fit ($Sa = 0.18$ and $Ta = 0.18$) predicted positions for the same time instants.

References

[1] Schaub H, Junkins JL. Analytical Mechanics of Space Systems, Fourth Edition. *Analytical Mechanics of Space Systems, Fourth Edition*. Epub ahead of print 1 April 2018. DOI: 10.2514/4.105210.

[2] Shih CC, Buchanan HJ. The drag on oscillating flat plates in liquids at low Reynolds numbers. *J Fluid Mech* 1971; 48: 229–239.

[3] Loth E. Drag of non-spherical solid particles of regular and irregular shape. *Powder Technol* 2008; 182: 342–353.

[4] Lamb H. *Hydrodynamics*. 6th ed. Dover Pub, https://www.cambridge.org/us/academic/subjects/mathematics/fluid-dynamics-and-solid-mechanics/hydrodynamics-6th-edition?format=PB&isbn=9780521458689 (1932).

[5] Brennen CE. A Review of Added Mass and Fluid Inertial Forces, https://resolver.caltech.edu/CaltechAUTHORS:BREncel82 (1982, accessed 5 December 2021).

[6] Zastawny M, Mallouppas G, Zhao F, et al. Derivation of drag and lift force and torque coefficients for non-spherical particles in flows. *International Journal of Multiphase Flow* 2012; 39: 227–239.
