# Supplementary material for: Omnidirectional propulsion in a metachronal swimmer
Source: PLoS Comput Biol. 2023 Nov 17;19(11):e1010891. doi: 10.1371/journal.pcbi.1010891 (PMC10697607; doi:10.1371/journal.pcbi.1010891)
Supplement: Attachment — Submitted filename: 1-ReviewerComments_HerreraByron.docx [file pcbi.1010891.s008.docx]

**Response to Reviewer #1**

*(1) A critical piece of information gathered from the experimental snapshots is beat frequency, which then enters the kinematic computational model. How is this data converted to a time-continuous series? I see the fit in supplemental fig.3 but there is no information about how this first is performed, what it captures and what it misses, if there is any filtering etc.*

We agree that this is a critical point and should be made clearer. We have added text to the supplementary material to clarify how we convert the measured frequency, which is resolved only at the timescale of the beat cycle, to a higher time resolution *via* a fitted spline (see lines 118 to 131 in the revised supplementary material). We also discuss the pros and cons of this approach: it provides a (more or less) continuous input for the model, but smooths out rapid oscillations in the frequency timeseries (as noted in the response to comment (2) below). An alternate approach might have been to introduce step changes to the model: that is, hold the frequency constant for the approximate duration of the corresponding beat cycle, then discretely change the frequency. However, this would be unphysical. Our definition of the frequency is $f=1/T$ where $T$ is the observed time interval between the start of one power stroke and the start of the next. This measured frequency is discrete as an artefact of the definition; however, the motion of each ctene is smooth. We have prioritized keeping the ctene motion smooth in the model over exact fidelity to the frequency. In the biological system, rapid changes in beat frequency such as those observed here do not lead to “jerky” swimming trajectories; changes in swimming speed and direction are smooth and continuous. Because our model does not take into account the actual flow interactions, or the elasticity of the ctenes and mesoglea below, a jerky prescribed frequency would lead to a jerky swimming trajectory—which is not what we observe in the biological system. We have therefore chosen to smooth the input frequency in this way.

*(2) On that note, the data in supplementary fig.3 is unclear: there are two sets of points and two curves. Which curve fits which data? Are the data oscillating between the upper sets and the lower sets of dots (in which case the curves miss the rapid oscillations)? Or are the data separately the upper and lower sets (in which case the fits seem systematically shifted).*

We agree—the two sets of points for ctene rows 4 and 5 were not clearly delineated. We have changed Fig 3 so that the frequencies on the two tracked rows now show in different subplots. The fitted curves do miss what appear to be rapid oscillations in the frequency, but we believe that those oscillations are an artefact of our definition of frequency (see response to comment (1) above) and that the strong smoothing is appropriate as it reproduces features of the swimming that (in the biological system) are likely driven by interactions in the flow and/or the deformations of the ctenes and mesoglea.

*(3) There seem to be clusters or groups of blue points In figure 3 [NOTE: now Fig 7] of the main text (the MAP diagram). Do these correspond to physically related conditions? One could almost squint and make out 4 straight lines around which most of the points are clustered. Could these correspond to the different modes? If so, could the modality be somehow ‘scaled’ out to collapse the data?*

These clusters/lines do indeed correspond to physically related conditions—they are specific values of $\Delta f\equiv f_{out}-f_{in}$. The lowest value of $\Delta f$ in our simulations is $\Delta f=2Hz$, which corresponds to the quadratic-appearing upper bound in Fig 7 (formerly Fig 3); as $\Delta f\to0$, we would see $\bar{R/L}\to\infty$ (that is, swimming in a straight line). In this case, as $\Delta f$ becomes smaller compared to $f_{out}$ and $f_{in}$, we see increases in both $\bar{V}$ and $\bar{R/L}$. In response to your comment, we attempted to scale $\bar{V}$ by both $\bar{f}$ (the average of $f_{in}$ and $f_{out}$) and $\Delta f$ ($f_{out}-f_{in}$). However, this did not yield any additional clarity. Instead, we added a figure (Fig 8) which breaks out all four turning modes and clearly shows the clustering by $\Delta f$.

*(4) Caption for figure 3 lists (A) and (B) which do not appear in the figure.*

Thank you very much for catching this. We have adjusted the text of this caption (now Fig 7).

*(5) While the authors do a good job of clarifying what is not captured in this minimalist model (e.g. hydrodynamic interactions), some of the modeling choices could use more justification. For example, why the choice of the oscillating plate rather than a hinged ‘flapping’ plate which might seem more natural and perhaps simpler to model (i.e, the red dashed line as the plate instead of the green solid line in Fig. 9D [NOTE: now Fig 5])?*

Thank you for this thoughtful comment. Our choices are in large part driven by our past and present interest in spatiotemporal asymmetry. Spatial asymmetry, which can be quantified in many ways, is straightforwardly illustrated by a simple change in the flow-normal area of the paddle (higher on the power stroke, lower on the recovery stroke). This change in area would not take place in a simple hinged flapping plate, though it could be achieved with a more complex two-bar linkage as has been used in other models (e.g. Alben et al 2010, Ford and Santhanakrishnan 2019). However, the two-bar linkage seemed to us to be less representative of the general case (and the simplest definition) of spatial asymmetry. At low Reynolds numbers, a spatially symmetric stroke will never produce net thrust due to the time-reversibility of the flow; therefore, our model (which was developed to investigate the scaling of drag-based, metachronal propulsion from low to intermediate Re) must have the capacity for spatial asymmetry. Our choice was also driven by the availability of empirical drag coefficients for flat plates at intermediate Reynolds number—the non-independence of drag from Re is a key piece of the problem and a large part of what makes our approach novel. We have added some text in the manuscript to clarify our position (see lines 237 to 242).

*(6) A key discussion element is the ‘mode’ of turning. Would it be possible to sketch a cartoon that clearly demarcates the 4 modes that are repeatedly discussed throughout the paper?*

Yes—we are happy to add a figure that more clearly explains the four modes to ensure that the reader understands our intent. Please see the new Fig 6, which has four panels schematically showing the four turning modes.

*(7) A comparison between and discussion around the measured and simulated trajectories (such as in supplementary fig. 4) might be apt in the main text.*

We agree that it is important to highlight what the model can reproduce (in terms of features of observed trajectories) and what it cannot, including its broader limitations. We have added a sentence to the final paragraph of the manuscript (under Materials and Methods, with a new section subheading “Model Performance and Validation” to summarize some of these differences, and refer the reader to the supplement for more details.

**Response to Reviewer #2**

*(1) Some morphometric parameters in Table 1 and Fig. 2 were hard to understand. For example, which rows did the sagittal row and tentacular row refer to, respectively? It is better to give straightforward schematic diagrams to illustrate the sagittal and tentacular plane and all the morphometric parameters for easier read.*

We agree, it is important to clarify what is meant by the morphological terms. Here, “sagittal rows” are the rows adjacent to the sagittal plane, and “tentacular rows” are the rows adjacent to the tentacular plane. We feel that adding specific labels for these rows to Fig 2A (now Fig 3A) would crowd the figure to an extent that would outweigh any additional clarity; however, we have added text to the caption of Fig 2 (now Fig 3) and to Table 1 to ensure that this definition is clear. We also identified two other parameters defined in table 1 which were not explicitly specified in the accompanying figure ($l$ and $\delta$), and have added these to Fig 3D (formerly Fig 2D).

*(2) In the Turning performance part, the authors mentioned that “In the first strategy (mode 1), two adjacent rows … and the two opposite rows … while the remaining four rows are inactive.” However, the “two adjacent rows” and “two opposite rows” have not been identified.*

To clarify the four modes, we have added a schematic figure (Fig 6). While this does not cover all possible permutations that may be used by the animals (since each body quadrant, containing two rows, may be excited at a different frequency), but it is consistent with what we observe in terms of turning maneuvers in free swimming.

*(3) In the Omnidirectionality part, the authors stated that “in our observations, animals swam freely (without external stimuli)” and recorded the trajectories of the ctenophore in Fig 4 [NOTE: now Fig 9]. So, the motion of the ctenophore in space should be random. However, as shown in Fig 4A [NOTE: now Fig 9A], the ctenophore mainly moved along the X axis and hardly along the Z axis, which resulted in the elliptical gray cloud. Why did the ctenophore tend to move along the X axis rather than the Z axis?*

In our data, we do observe movement in space that is random. However, to more clearly compare the trajectories to one another, we reorient the trajectories so that each animal occupies the same position and orientation at time $t=0$: we place the approximate center of mass (the midpoint between the tentacular bulbs) at the origin, and align the tentacular plane with the X-Y plane. The animals cannot swim sideways—only forward and backward. Our dataset only contains animals with a nonzero initial velocity, because we only record sequences where the animal freely swims through the field (volume) of view. Put another way: all measured trajectories have a nonzero initial velocity, and we have reoriented the trajectories so that this initial velocity points along the X axis. This results in a motor volume that is stretched along the X axis—but roughly symmetric in the Y and Z axes, which supports our assertions regarding maneuverability and omnidirectionality. We have modified the caption of Fig 9 to make this point more clear, and have added a cartoon of the ctenophores’ initial position to further clarify.

*(4) What is the physical meaning of BL/s as the unit of the average speed?*

Because animals in our dataset vary somewhat in size (from 6.34 to 11.56mm), we normalize the swimming speed by dividing by $L_{B}$ (such that the average speed is measured in body lengths per second rather than absolute speed), as is common in studies of locomotion.

*(5) The typos should be correct. For example, “via” in line 57 should be written in upright form and “30×30×30 〖mm〗^2” should be “30×30×30 〖mm〗^3”.*

Thank you for pointing these out. We have corrected them in the revised manuscript.

**Response to Reviewer #3**

*1. The title claims that “metachronal coordination enables omnidirectional swimming”. As we know, metachronal coordination, as a cross-scale phenomenon, is ubiquitous [Integr. Comp. Biol. 2021, 61, 1674-1688.]. It has been verified that metachronal motion can extremely enhance the propulsion ability of natural cilia [PNAS 2013, 110, 4470-4475.]. However, the observations in this paper suggest that omnidirectional swimming is mainly due to the combination of (i) different swimming modes (according to actuated rows and adjustable beating frequency to distinguish) and (ii) the flexible forward/reverse swimming of ctenophores. By providing symmetric (mode 4) or asymmetric (mode 1-3) propulsion along the body axis, ctenophores can control themselves to go straight or turn. Even if the propulsive forces are provided from ciliary beating and metachronal coordination, however, the omnidirectional swimming is actually realized by the smart swimming strategies of ctenophores, and is not directly related to metachronal coordination. In particular, when the authors use a reduced-order analytical model that ignores hydrodynamic interactions among propellers, the summary (“metachronal coordination enables omnidirectional swimming”) becomes weird and incomprehensible. Please explain this issue.*

The reviewer brings up an excellent point—it is not necessarily metachrony that enables the high degree of maneuverability seen in ctenophores, but rather their body plan and the independence of the ctene rows. The metachronal coordination does smooth the propulsive forces (see Herrera-Amaya et al 2021 ICB), and generally contributes to the equivalent forward-backward swimming capability (which exceeds that found in most other swimmers). However, the direct connection between metachrony and omnidirectional swimming that is implied by the title is certainly not quite right. Our goal with the title was to broaden the appeal of the manuscript beyond readers with a niche interest in this specific model system. However, we agree that the current title implies an assertion that is not supported by the manuscript itself. We suggest a new and slightly altered title of “Omnidirectional propulsion in a metachronal swimmer”, which does not imply a direct relationship between the maneuverability and the metachronal coordination (but retains descriptive value for those who study metachrony and may be interested in the work for this reason).

*(2) The authors indicate that 3D high-speed videography is first used to observe ctenophores and past works usually adopt a 2D imaging system. Surely, the discovery of four types of 3D swimming modes is impressive. But does this system have other advantages? It will be great if the author can list a table to compare the experimental conclusions and technical limitations of some previous 2D imaging systems, and which ones are well resolved in the 3D imaging system of this article. And in the “Conclusion” section, an appropriate discussion of this system’s potential for follow-up research is also expected.*

While we are the first to explicitly study ctenophore turning in 3D, we are of course not the first to use three-dimensional-resolved high speed videography to study animal locomotion (see [J Exp Biol (1986) 126 (1): 479-497, Biol Open (2016) 5 (9):1334-1342, J Exp Biol (1998) 201 (5):655-672, AIAA Journal, Vol. 57, No.9, Sep 2019]). However, there is certainly potential to explore interesting follow-up questions using 3D resolved videography. We feel that the manuscript is already lengthy and that an additional table could clutter it, but we have added text to both the introduction (lines 87-90) and to the conclusion (lines 548-550) underscoring the potential of 3D videography to enable the pursuit of interesting questions. This is particularly relevant in light of the decreasing price points of workable high speed camera systems, which democratizes access to this kind of analysis.

*(3) Again, the authors repeatedly emphasize the importance of the metachrony of ctenophores, please give some related qualitative/quantitative descriptions of their motion parameters, e.g., antiplectic or symplectic, the phase difference of neighboring cilia in the metachronal coordination, etc.*

Because the power stroke can reverse directions, coordination in ctenophores may be either symplectic or antiplectic; however, it is most frequently antiplectic, and we have added this to the manuscript (line 64). The phase lag is discussed in the methods section (under the new subheading “Model Performance and Validation”). The experimental measurements discussed in this manuscript cannot provide a detailed view of the variability of phase lag, because they are spatially resolved at the scale of the body rather than the scale of the ctenes (to enable visualization of swimming trajectories). This is also the case for several other kinematic parameters that are directly related to the metachronal coordination and/or motion at the scale of the ctene: we do not have the spatial resolution to draw these parameters directly from the same experimental dataset from which the swimming trajectories are measured. We therefore choose a single representative value of phase lag ($P_{L}=13.2\%$) and several other variables based on measurements from our previous work (Herrera-Amaya et al 2021 ICB). We note that we have already explored the effects of varying phase lag on the model results in this previous study; we choose not to do so here, in order to limit the parameter space to a reasonable landscape.

*(4) Because of the gap between the mathematical model and real biological swimmers, correspondingly, there exists a gap between the computational simulation results and the experimental results. The authors should comment on this, especially when the authors want to throw out the conclusions such as “The model results confirm that ctenophores’ metachronal rowing platform is highly maneuverable and agile, with performance limits that may extend beyond our experimental observations (lines 183-185)”.*

We agree that this is an important acknowledgment to make, and the model is extensively compared to experimental results in the supplemental material. However, in response to feedback from reviewers here and elsewhere, we have concluded that this topic should certainly be raised in the main text as well. We have added a paragraph at the conclusion of the methods section summarizing the discussion in the supplement, with a new subheading titled “Model Performance and Validation.”

*(5) It is worth noting that when the authors would like to emphasize that the swimming performance is excellent, please make the conclusion more objective and intuitive. For example, lines 167-168 “still a comparatively sharp turn, carried out at 71% of the maximum recorded straight-line swimming speed”. The authors want to use this to indicate that ctenophores keep both high agility and maneuverability. But it seems that the maximum speed is from another individual, and how could the authors rate that 71% is an excellent indicator? Maybe there are some comparisons to other swimmers’ turning movements?*

This is a fair point. Our use of “maximum recorded… swimming speed” was motivated by the fact that our dataset comes from freely swimming animals, which are not stimulated in any way. It is therefore difficult to draw conclusions about the limits of performance, and our comparisons are necessarily constrained; we cannot force one individual to perform exactly the same maneuver as another, so we cannot directly compare between animals, nor can we induce a single animal to perform both straight swimming and turning. We must therefore use the limits of the overall dataset as rough proxies for the extremes of performance. We note that we have expressed the swimming speed in body lengths per second to facilitate comparisons across animals. However, we agree that it is difficult to intuitively grasp the relatively excellent performance of ctenophores without also comparing to other organisms. We have added a new line of text (lines 372-376) that references similar data for a much faster swimmer, the whirligig beetle, which places our results in some context (in this study, the authors also consider the maximum of the entire dataset, rather than the maximum of a particular individual, because of the same issues regarding the full exploration of the performance landscape). We also note that our model shows similar performance and is based on the “average” swimmer from our experimental dataset.

*(6) I understand the authors want to put some technical details in the “Methods” section for detailed analysis and discussion. But please briefly describe the used methods in the main text, and refer to the specific location in “Methods”. The coherence and logic of various parts in the main text are poor, thus the readability is greatly affected. Please reorganize the manuscript. Also, some tiny problems exist, for example, line 249 directly indicates Figure 7B, but Figure 6 is still not described at this time. The authors do need to make the manuscript clear.*

We agree, and initially placed the methods section at the end of the manuscript to comply with suggested guidelines for PLOS journals (see these guidelines at <https://journals.plos.org/ploscompbiol/s/submission-guidelines>). However, the bulk of the study’s contribution is the comparison between the experimental results and the model, as well as the use of the model to explore the performance landscape in ways that are not possible with animal experiments. Because of this, and because of this comment and others, we believe that placing the methods in the middle of the manuscript (rather than at the end) will be clearer and more readable. We have made the case for this organization in the cover letter for our revised manuscript, and hope that the editor will consider allowing an exception to the suggested formatting for the sake of clarity.

**Response to Reviewer #4**

*(1) Line 112 spell out Bolinopsis (first time used in Intro)*

Thank you; this has been corrected.

*(2) Line 333 and Blue?*

We are not sure to what this comment may refer; the caption of Fig 7 (now Fig 2) states that the blue line is the path of a tentacular bulb. There is no blue triangle. The green and red triangles referenced are the lightly shaded areas of the figure, indicating the starting and ending position of the animal.

*(3) Fig. 3 – Should distinguish the different modes in the figure. Cannot interpret what each blue scatter line represents.*

Thank you for this comment. We have revised Fig 3 (now Fig 7) to be more clear, and have added an additional figure (Fig 8) that shows the same data, broken out according to the turning modes (with schematic labels) and clearly identifying that the clusters of points correspond to different values of $\Delta f$. We have also added some text that further discusses the differences between the modes (lines 403-407).

*(4) Lines 171-185 – The simulation results description in this paragraph does not add anything to the experimental observations. In experiments Bolinopsis is able to have high maneuverability while being agile. Model just confirms that Bolinopsis is able to do this using its metachronal ctenes. That doesn’t add anything. The ctenes are obviously able to do this.*

We respectfully disagree; the model places the experimental data within a larger parameter space by exploring limits that are not observed in the experiments. Because our experimental dataset consists of freely swimming animals, which are not externally stimulated (unlike similar experiments in other organisms, which provoke e.g. an escape response) it is unlikely that we are exploring the extremes of behavior. Furthermore, the experiments only patchily cover the range of the possible values for each kinematic variable. The reduced-order model, by contrast, can explore the full range of possibility. While Fig 3 shows the extent of the tested model cases, we highlight in the text two notable observations: 1) the model captures approximately the same maximum maneuverability as seen in the animal observations ($\bar{R/L}=0.08$), and 2) it points to the possibility that the agility we observed in the swimming animals may not have reached the peak capacity of the locomotor system (since the model achieved 93% of the top swimming speed while still maintaining $\bar{R/L}<1$. We feel that these observations both underscore the validity of the model (as it replicates the animals’ behavior) and also illustrates how reduced-order model can be a valuable tool to explore the limits of performance when experimental data are limited to undriven behavior.

*(5) Fig. 4 – it would be very helpful to place ctenophore icons showing the orientation of bolinopsis at different locations in the MV. Otherwise, very difficult to interpret the MV.*

Thank you for this excellent suggestion. We have added a sketch to Figure 4 (now Fig 9) showing the initial orientation of the ctenophores, which also helps to underscore our approach of rotating each trajectory to the same initial position. The motion is highly three-dimensional, so it is difficult to add a sketch of the animal in an accurate orientation at other points along the trajectory; we also feel that this would clutter the figure. However, we agree that the addition of an initial orientation icon is quite helpful.

*(6) Fig. 5 – display different simulated modes with different colors or line styles.
Model predicts that mode 1 is for high maneuverability while modes 2 and 3 allow for faster agility. You should use kinematic data to confirm these predictions. In other words, you should report the maneuverability and agility of each mode that you observed.*

This is a great suggestion—thank you. We have added colors to Fig 5 (now Fig 10) to distinguish between the three turning modes, and added a table (Table 6) which lists the values of $\bar{R/L}$ and $\bar{V}$ for each of the trajectories displayed in Fig 5 (now Fig 10).

*(7) Lines 240-243 – this belongs in the Discussion and should be expanded to discuss what the experimental work and model suggest about how ctenophores a achieve different maneuvering outcomes. This would also be the spot to bring in maximizing R/L by reversing inside ctenes as mentioned in lines 284-286.*

We agree that these small comments are perhaps more appropriate for the Discussion, but hesitate to waste the opportunity to highlight the implications of this result while it is fresh in the reader’s mind. We have chosen to leave these two sentences as-is, but expand upon this topic in the Discussion (see lines 534-542), which allows us to speculate further about which turning modes are most appropriate for which functional behaviors.

*(8) Line 256 should be in past tense “we explored”*

We have adjusted this phrase to be in the present tense, rather than the future tense (we note the remainder of the Results section is written in the present tense—not the past tense—and have maintained this usage for consistency).

*(9) Line274-277 – This is an interesting observation but needs to be quantified and reported in the Results. What as the mean and max R/L*

Our observation was intended to refer to the known morphology of ctenophores (see e.g. Tamm 2014 *Invert. Bio.*), and to highlight that the particular species we study here (*B. vitrea*) is a type of ctenophore which is known to have incomplete radial symmetry in terms of the length of the tentacular vs. sagittal rows as well as the presence of large lobes. It would be interesting to repeat the experiment with a cydippid species, which ostensibly drives fluid motion in the same way but achieves a greater degree of radial symmetry as its eight ctene rows are all the same length. We quantify the difference between forward and backward swimming by reporting $\bar{R/L}$ and $\bar{V}$ for the trajectories shown in Fig 5 (now Fig 10) (see new Table 6). We have also added the minimum achieved $\bar{R/L}$ and maximum achieved $\bar{V}$ to the caption of Fig 6 (now Fig 11).

*(10) Lines 284-286 – What is the support for this statement? Literature or your data.*

This statement stems from personal observation and consultation with colleagues (e.g. George Matsumoto from MBARI). We are happy to cite as such if desired.

*(11) Discussion should be expanded to discuss, based on your experiments and model, how ctene rows can be manipulated to achieve different swimming outcomes (see above). You should also discuss, based on what we know about how ctenophores use their apical organ to control ctene rows, what the what the potential limits are to modes of swimming. Can ctenophores independently control all ctene rows like you simulated in Fig. 6? What can they do?*

We have added a section to the Discussion (see lines 534-542) which explores this in more detail. We clarify again here that Fig 6 (now Fig 11) is a hypothetical case: because of the architecture of the statocyst and apical organ, ctenophores cannot control each row independently. Instead, they control each body quadrant, with a pair of ctene rows carrying a unique frequency. However, a bioinspired device or vehicle would not be subject to the same limitations and would therefore be able to occupy more of the performance landscape. The goal of Fig 6 (now Fig 11) is to explore this landscape.
